# Equation-derived SMI not generalizable for DXA-defined low muscle mass in Chinese adults: external validation study

**DOI:** 10.3389/fnut.2026.1887277

**Published:** 2026-09-22

**Authors:** Zhenchao Liu, Gudi Shi, Yuqian Ren, Yun Wang, Xinbo Zhao

**Affiliations:** 1 School of Pharmacy, Qingdao University, Qingdao, China; 2 Health Management Department, Shandong Medicine Technician College, Taian, China; 3 School of Basic Medicine, Qingdao University, Qingdao, China; 4 Health Management Center, Linyi People’s Hospital, Linyi, China; 5 Department of Endocrinology, Linyi People’s Hospital, Linyi, China

**Keywords:** anthropometric prediction equation, DXA, external validation, sarcopenia, skeletal muscle index

## Abstract

**Background:**

Anthropometric equations are commonly used to estimate appendicular skeletal muscle mass (ASM) when direct body-composition measurements are unavailable. However, it remains unclear whether skeletal muscle index derived from predicted ASM (SMI_pred) can validly identify low muscle mass using thresholds developed for dual-energy X-ray absorptiometry (DXA). We externally validated the Wen et al. anthropometric equation against DXA in Chinese adults undergoing routine health examinations.

**Methods:**

This single-center retrospective external validation study included 501 Chinese adults who underwent whole-body DXA between 2016 and 2022. The published equation was applied without coefficient updating to calculate predicted ASM (ASM_pred), and SMI_pred was calculated as ASM_pred divided by height squared. Validation included Pearson correlation, Lin’s concordance correlation coefficient (CCC), calibration, Bland–Altman agreement, and classification of DXA-defined low muscle mass by direct application of the Asian Working Group for Sarcopenia 2019 sex-specific DXA cut-offs. Simple linear recalibration was evaluated using repeated 10-fold cross-validation as a secondary analysis.

**Results:**

ASM_pred was strongly correlated with DXA-measured ASM (Pearson *r* = 0.892, *95%CI* = 0.873–0.909; CCC = 0.862), but systematically overestimated ASM, with a mean bias of 1.25 kg and 95% limits of agreement from -3.14 to 5.64 kg. Its calibration slope was 0.853 (*95%CrI* = 0.815–0.891). SMI_pred showed lower correlation and concordance with DXA-measured SMI (Pearson *r* = 0.756, *95%CI* = 0.716–0.791; CCC = 0.705), a mean bias of 0.457 kg/m^2^, 95% limits of agreement from -1.19 to 2.10 kg/m^2^, and a calibration slope of 0.738. The difference between the ASM and SMI correlations was 0.136 (bootstrap *95%CI* = 0.111–0.164; *P* = 0.0002). Direct application of AWGS 2019 DXA cut-offs to SMI_pred yielded a sensitivity of 0.211 and specificity of 0.963, missing 97 of 123 DXA-defined low-muscle-mass cases. Cross-validated recalibration reduced prediction error but did not eliminate individual-level disagreement.

**Conclusion:**

The Wen equation preserved strong association with DXA-measured absolute ASM but showed systematic bias and limited individual-level agreement. Performance deteriorated after derivation of SMI, and direct transfer of DXA-based AWGS thresholds to SMI_pred resulted in substantial under-detection of low muscle mass. Neither the original nor the locally recalibrated equation should be used as a stand-alone substitute for DXA when classification depends on fixed muscle-mass thresholds. Any use within a multivariable screening pathway requires separate validation of the complete screening rule.

## 1 Background

Skeletal muscle is essential for physical function, metabolic homeostasis, and overall health (1, 2). With aging, muscle mass, strength, and function progressively decline, potentially leading to sarcopenia (3). As a major geriatric syndrome, sarcopenia is associated with falls, disability, hospitalization, and mortality (4). Early identification of low muscle mass is therefore of substantial clinical and public health importance.

Dual-energy X-ray absorptiometry (DXA) is widely used as a reference method for appendicular skeletal muscle mass (ASM) assessment and is recommended in multiple guidelines (5–7). However, its use in routine practice and large-scale screening is limited by cost, accessibility, and operational requirements (8). Anthropometric prediction equations therefore remain attractive as simple and scalable alternatives in health examinations, primary care, and epidemiological studies. Several anthropometric equations have been developed to predict ASM in Chinese populations, but their external validity and clinical transportability remain insufficiently established, particularly for threshold-based classification. The equation proposed by Wen et al. (9) is widely known and has been used in datasets lacking DXA measurements. However, good performance in a derivation sample does not guarantee acceptable agreement, calibration, or classification accuracy in independent populations. This concern was recently raised by Liu et al. (10), who questioned the use of formula-derived muscle indices without external validation.

Importantly, when predicted ASM is further indexed by height^2^ to derive skeletal muscle mass index (SMI) and subsequently used to classify sarcopenia severity, any initial prediction error may be amplified, thereby affecting the identification of pre-sarcopenia and sarcopenia. Although indexation helps account for body size differences, it may also alter the error structure, such that systematic bias in ASM prediction becomes more pronounced at the SMI level. Therefore, even when a prediction equation performs well for continuous ASM estimation, its measurement error may be amplified after height-indexing and may not translate into adequate discriminative accuracy for SMI stratification or sarcopenia classification. From a methodological perspective, reporting correlation coefficients alone is insufficient to establish interchangeability at the individual level, because correlation reflects linear association rather than absolute agreement or concordance. By contrast, concordance and agreement analyses, calibration assessment, and evaluation of classification performance for clinically relevant stratification can more accurately reflect the real-world validity and utility of anthropometric prediction equations. Unfortunately, independent external validation evidence for these equations in Chinese adults undergoing health examinations remains limited, particularly studies that simultaneously assess performance across multiple dimensions, including correlation, agreement, calibration, and discriminative ability for clinically relevant stratification. Addressing this evidence gap is crucial to prevent systematic misclassification and inappropriate clinical decision-making in large-scale sarcopenia screening. Existing evidence has also rarely compared predicted ASM/SMI with DXA-measured ASM/SMI across different muscle-mass and strength states, and there is limited analysis of whether these predicted indices can distinguish normal status, low muscle mass with normal grip strength, and low muscle mass with low grip strength.

Therefore, we performed an external validation study against DXA in Chinese adults undergoing health examinations to assess the transportability of a published anthropometric equation. Accordingly, the aim of the present study was not to develop a new prediction model but to test whether a published equation could be transported to an independent sample and used in place of DXA for clinically relevant threshold-based decisions without coefficient updating. Our primary focus was whether the derived SMI could support threshold-based identification of DXA-defined low muscle mass. The principal methodological contribution of this study was therefore the evaluation of threshold transportability and individual-level classification performance in an independent population, rather than another assessment of conventional prediction accuracy or correlation alone. We consequently prioritized agreement, calibration, and low-SMI classification performance alongside correlation, with broader sarcopenia-stage analyses treated as secondary.

## 2 Methods

### 2.1 Study population

This was a single-center retrospective external validation study of a published anthropometric ASM equation using a previously established local database of adults undergoing routine health examinations. The study population was drawn from adults who underwent health examinations and completed dual-energy X-ray absorptiometry (DXA) at Linyi People’s Hospital between January 2016 and December 2022. Study data were retrieved and extracted from the hospital information system (HIS), electronic medical records (EMRs), and the DXA database.

Adult participants who underwent health examinations during the study period and completed whole-body dual-energy X-ray absorptiometry (DXA) scans were first identified. Candidate participants were then manually reviewed using electronic medical record (EMR) data to determine whether they met the predefined inclusion and exclusion criteria. The present study constituted a further analysis based on a previously established database related to sarcopenia screening and diagnosis. Because the present cohort was assembled independently from routine health-examination participants at Linyi People’s Hospital and did not reuse any derivation data from Wen et al., this study provided an independent external validation setting for assessing transportability to relatively healthy Chinese adults undergoing health examinations. No participants from the derivation dataset of Wen et al. were included, and no model coefficients were updated prior to validation. The study protocol was approved by the Ethics Committee of Linyi People’s Hospital (approval No. 202504-H-010). Given that this is a retrospective study using existing health examination and clinical records, no interventional procedures were performed. Written informed consent was not required in accordance with the approval of the Ethics Committee. All inclusion and exclusion criteria were applied according to the predefined rules during data preparation to ensure accuracy and consistency. Reasons for exclusion during the participant selection process (e.g., missing key variables and diseases that may affect skeletal muscle mass) were handled and documented prior to statistical analysis.

#### 2.1.1 Definition of sarcopenia status

This study referred to the AWGS 2019 (Asian Working Group for Sarcopenia 2019) cut-off values for low muscle mass and low muscle strength (6). For the purposes of this study, participants were classified into three categories: normal, low muscle mass with normal grip strength, and low muscle mass with low grip strength. Sarcopenia status was defined according to the AWGS 2019 criteria for Asian populations. Low muscle mass was defined using sex-specific DXA-derived SMI cut-offs (men: 7.0 kg/m^2^; women: 5.4 kg/m^2^), and low muscle strength was defined as handgrip strength < 8 kg in men and <18 kg in women. For the study-specific three-category analysis, participants were categorized as (i) normal muscle mass and strength, (ii) low muscle mass with preserved handgrip strength, and (iii) low muscle mass with low handgrip strength. For brevity, the second category is referred to descriptively as the “low-muscle-mass-only” group rather than as a formal AWGS 2019 diagnostic category. In this manuscript, “pre-sarcopenia” is used as a study-specific descriptive label for low muscle mass with preserved handgrip strength and should not be interpreted as a formal AWGS 2019 diagnostic category. No participant had normal DXA-derived SMI combined with low handgrip strength; therefore, these three mutually exclusive categories included all 501 participants.

#### 2.1.2 Inclusion criteria

Participants were included if they met all of the following criteria: (1) age ≥18 years; (2) underwent whole-body DXA and had contemporaneous anthropometric and handgrip assessments within the same health-examination episode; (3) had complete anthropometric data, including age, sex, height, and body weight; (4) had handgrip strength measurements available; (5) participants were considered relatively healthy on the basis of the absence of documented EMR diagnoses of conditions known to markedly affect muscle mass, although residual undiagnosed or subclinical comorbidity could not be fully excluded in this retrospective health-examination cohort.

#### 2.1.3 Exclusion criteria

Participants were excluded if they met any of the following criteria: (1) known diseases that could substantially affect skeletal muscle mass, including diabetes mellitus, malignancy, liver cirrhosis, severe cardiovascular or cerebrovascular disease, thyroid dysfunction, and rheumatic or autoimmune disease, identified primarily from contemporaneous EMR diagnoses; because exclusionary conditions were ascertained mainly from EMR diagnostic information, undocumented or undiagnosed comorbidities could not be completely ruled out; (2) long-term use of medications that might affect muscle mass, such as glucocorticoids or sex hormones; (3) acute infection, major trauma, major surgery, or disuse-related secondary muscle atrophy (e.g., prolonged bed rest or limb immobilization with plaster fixation) within the previous 3 months; or (4) incomplete medical records or missing key variables that precluded valid analysis.

Because the predefined exclusions removed several chronic conditions that may alter muscle mass, the target population of this validation was restricted to relatively healthy adults undergoing health examinations rather than an unselected clinical population. Accordingly, the present analysis evaluates transportability within this restricted setting and does not establish performance in patients with diabetes, malignancy, cirrhosis, severe cardiovascular disease, or other muscle-wasting conditions.

### 2.2 Data collection and variable definitions

All data from eligible participants were extracted in an anonymized manner. Data sources included the hospital EMR system, the health examination system, and the DXA database. Data extraction was independently performed by uniformly trained researchers and cross-checked to ensure accuracy and completeness.

The variables collected in this study included the following categories:

#### 2.2.1 General demographic and anthropometric data

Variables collected included sex, age, height, and body weight. Age was defined as the actual age on the date of the DXA examination. Height and body weight were obtained from contemporaneous health examination records or anthropometric information attached to the DXA report. Body mass index (BMI) was calculated as body weight (kg)/height (m)^2^.

#### 2.2.2 Skeletal muscle-related indices

DXA-measured appendicular skeletal muscle mass (ASM)

Whole-body body composition was assessed using DXA (Lunar Prodigy, GE Healthcare, Madison, WI, USA) according to standardized whole-body scanning protocols. DXA acquisition was performed using standardized whole-body scanning protocols, and device calibration/quality control followed the manufacturer’s recommendations. Appendicular lean mass values for the limbs were obtained from the device software, and appendicular skeletal muscle mass (ASM) was derived as their sum according to standard DXA-based practice.

DXA-measured skeletal muscle mass index (SMI)

SMI was calculated as ASM (kg)/height (m)^2^.

Anthropometry-predicted appendicular skeletal muscle mass (ASM_pred)

Appendicular skeletal muscle mass was estimated using the anthropometric prediction equation proposed by Wen et al. for the Chinese population ^9^: ASM_pred = 0.193 × weight (kg)+0.107 × height (cm)−4.157 × sex−0.037 × age (years)−2.631, where sex was coded as 1 for men and 2 for women. The anthropometric prediction equation by Wen et al. was applied as published without re-estimation or re-calibration in the current cohort to evaluate transportability.

Anthropometry-predicted skeletal muscle mass index (SMI_pred)

SMI_pred was calculated as ASM_pred (kg)/height (m)^2^.

#### 2.2.3 Muscle strength

Handgrip strength was measured using a standard handgrip dynamometer (Xiangshan, China) according to the routine operating procedures of the health examination center. The maximum handgrip strength value (kg) was recorded for analysis. Handgrip strength results were used in combination with skeletal muscle mass indices to stratify sarcopenia status.

### 2.3 Statistical analysis

All analyses were performed using R (version 4.5). The primary objective was external validation of the published equation against DXA. Primary analyses therefore focused on: (1) association and concordance between predicted and DXA-measured ASM/SMI using Pearson’s r and Lin’s concordance correlation coefficient (CCC), each reported with 95% confidence intervals (CIs); (2) agreement using Bland–Altman analysis, including mean bias and 95% limits of agreement, with proportional bias additionally assessed by regressing the paired difference on the paired mean, differences were calculated as the predicted value minus the corresponding DXA-measured value; therefore, a positive bias indicates overestimation by the equation; (3) calibration of predicted ASM/SMI against DXA-measured values; and (4) classification performance of SMI_pred for DXA-defined low muscle mass using AWGS 2019 cut-offs, including sensitivity, specificity, positive predictive value (PPV), negative predictive value (NPV), accuracy, and Cohen’s kappa, all reported with 95% confidence interval. We deliberately applied the conventional DXA-based AWGS cut-offs directly to SMI_pred because this reflects a common real-world analytic practice in datasets lacking direct body-composition measurements. Secondary regression and subgroup analyses were considered exploratory. Exploratory analyses of the three sarcopenia-related categories were conducted using multinomial logistic regression with a softmax formulation, with the normal category serving as the reference. The models evaluated whether predicted SMI (SMI_pred) was associated with category membership after adjustment for age, sex, and BMI. Model stability was assessed based on the number of outcome events relative to the number of estimated parameters. Because only 31 participants were classified as having sarcopenia, the multinomial models, models comparing sarcopenia with all other participants, and finely stratified sex-by-age analyses were considered potentially unstable. These analyses were therefore excluded from the primary results and are reported only as exploratory analyses in the Supplementary material, with sample sizes, event counts, and wide CIs explicitly presented. Calibration of continuous ASM and SMI was assessed using Bayesian linear regression models, with DXA-measured ASM or SMI as the dependent variable and the corresponding predicted value as the independent variable; calibration plots display binned observations together with a locally weighted smoothing (loess) curve, a linear fitted line, and the 45° line of identity. Because the ASM and SMI correlations were estimated from the same participants and were therefore statistically dependent, their difference was evaluated using a subject-level paired bootstrap with 10,000 resamples. In each resample, participants were sampled with replacement, both Pearson correlation coefficients were recalculated, and their paired difference was defined as Δr = r(ASM_pred, ASM) - r(SMI_pred, SMI). The percentile 95% CI and a two-sided bootstrap P value were obtained from the empirical distribution of Δr. Statistical significance was not inferred from overlap between the separate CIs for the two correlations. As a secondary model-updating analysis, simple linear recalibration was performed by regressing each DXA-measured value on the corresponding estimate derived from the original equation. The recalibrated estimates were defined as ASM_recalibrated = α_ASM + β_ASM × ASM_pred and SMI_recalibrated = α_SMI + β_SMI × SMI_pred, where α and β represent the recalibration intercept and slope, respectively. Apparent performance was first evaluated in the complete-case dataset used to fit each recalibration model. To reduce optimism, the recalibration procedure was additionally evaluated using repeated 10-fold cross-validation. In each repetition, the recalibration coefficients were estimated exclusively in the training folds and then applied to the corresponding held-out fold. For each participant, the out-of-fold predictions obtained across repetitions were averaged, and the resulting cross-validated predictions were pooled to calculate mean bias, 95% limits of agreement, mean absolute error (MAE), root mean squared error (RMSE), Lin’s concordance correlation coefficient (CCC), and calibration intercept and slope. Ten-fold cross-validation was repeated 100 times using prespecified random seeds of 20250308 for the ASM analysis and 20250309 for the SMI analysis. Calibration intercepts and slopes were estimated from these models and are reported as posterior medians with 95% credible intervals (CrIs). Bayesian linear regression models with weakly informative priors were used for continuous calibration analyses; model diagnostics are provided in the Supplementary material. The Bayesian framework was prespecified to obtain posterior distributions for the calibration intercept and slope and to quantify their uncertainty relative to the ideal calibration values of 0 and 1. The priors, convergence criteria, effective sample sizes, R-hat statistics, and posterior predictive checks are reported in the Supplementary material. A complete-case analysis was performed because all variables were required for equation application and outcome definition. Categorical variables are presented as counts and percentages, and continuous variables as mean ± standard deviation. Group comparisons were performed using one-way ANOVA or Welch’s ANOVA for continuous variables, and the chi-square test or Fisher’s exact test for categorical variables, as appropriate. All tests were two-sided, and *P* < 0.05 was considered statistically significant. Exact *P* values are reported to three decimal places, except that values below 0.001 are reported as *P* < 0.001. Frequentist uncertainty intervals are denoted as 95% CIs, whereas Bayesian posterior intervals are denoted as 95% CrIs. (Supplementary material includes multicategory analyses, sex-stratified discrimination analyses, decision-curve analyses, and Bayesian model diagnostics)

## 3 Results

### 3.1 Participant characteristics and prevalence of sarcopenia status

A total of 501 adults undergoing health examinations were included in the final analysis. The mean age was 51.23 ± 14.57 years and the mean BMI was 24.65 ± 3.87 kg/m^2^. The mean DXA-measured ASM and SMI were 17.71 ± 4.69 kg and 6.54 ± 1.19 kg/m^2^, respectively, whereas the corresponding predicted values were 18.96 ± 4.90 kg and 6.99 ± 1.21 kg/m^2^. According to the AWGS 2019 criteria, 378 participants (75.45%) were classified as normal, 92 (18.36%) as pre-sarcopenia, and 31 (6.19%) as sarcopenia. Overall, 123 participants (24.55%) had DXA-defined low SMI. Baseline characteristics according to sarcopenia status are shown in Table 1.

**TABLE 1:** Participant characteristics according to DXA-defined muscle-mass and handgrip-strength status [mean (SD)/median (IQR)].

Group	n	Age (years)	Height (cm)	Weight (kg)	BMI (kg/m2)	ASM (kg)	ASM_pred (kg)	SMI (kg/m2)	SMI_pred (kg/m2)	Handgrip (kg)
Normal	378	50.98 ± 13.32	163.74 ± 9.24	68.62 ± 13.82	25.45 ± 3.65	18.71 ± 4.58	19.41 ± 5.09	6.88 ± 1.04	7.13 ± 1.23	31.50 (16.50)
Pre-sarcopenia	92	46.95 ± 15.76*	162.31 ± 7.82	58.47 ± 9.95*	22.15 ± 3.22*	14.65 ± 3.47*	17.60 ± 3.91*	5.50 ± 0.90*	6.61 ± 1.01*	30.05 (14.42)
Sarcopenia	31	66.94 ± 15.54*#	163.01 ± 8.01	59.51 ± 11.85*	22.37 ± 4.18*	14.64 ± 3.92*	17.52 ± 4.29	5.45 ± 1.13*	6.52 ± 1.26*	16.50 (8.40)*^#^
*Test statistic*		19.12	1.17	35.86	40.71	51.35	8.36	93.65	10.86	54.62
*P values*		<0.001	0.315	<0.001	<0.001	<0.001	<0.001	<0.001	<0.001	<0.001

*P < 0.05 vs. normal; ^#^*P* < 0.05 vs. pre-sarcopenia.

Female sex did not differ significantly among the three groups. Among the continuous variables, all variables except height differed significantly across the three groups in the omnibus analyses. Participants with sarcopenia were older and had lower body weight, BMI, DXA-measured ASM, DXA-measured SMI, predicted ASM, predicted SMI, and handgrip strength than participants in the normal group. Compared with the normal group, participants with pre-sarcopenia also had lower body weight, BMI, DXA-measured ASM, DXA-measured SMI, and predicted SMI. In contrast, predicted ASM did not differ significantly between the normal and pre-sarcopenia groups after multiplicity adjustment (P = 0.052).

### 3.2 Primary validation of ASM_pred and SMI_pred against DXA

ASM_pred correlated strongly with DXA-measured ASM, whereas agreement and calibration analyses identified systematic overestimation and imperfect calibration. Compared with ASM_pred, SMI_pred showed weaker correlation, lower concordance, and more pronounced miscalibration relative to DXA-measured SMI, with substantial individual-level disagreement for both outcomes. In the overall sample, Pearson’s *r* was 0.892 (*95% CI* = 0.873–0.909) and CCC was 0.862 (*95% CI* = 0.843–0.878) for ASM_pred versus ASM. For SMI_pred versus SMI, Pearson’s *r* was 0.756 (*95% C*I = 0.716–0.791) and CCC was 0.705 (*95% CI* = 0.667–0.738).

The paired-bootstrap analysis confirmed that the correlation for ASM was statistically stronger than that for SMI. The observed difference was *Δr* = 0.136, with a bootstrap standard error of 0.014 and a *95% CI* of 0.111–0.164 (two-sided *P* = 0.0002). This comparison accounted for the dependence between the two correlations because both were estimated from the same 501 participants.

Bayesian linear recalibration showed that both ASM_pred and SMI_pred remained strongly associated with their corresponding DXA measurements but were imperfectly calibrated. Based on the posterior medians, the recalibration equations were ASM_recalibrated = 1.537 + 0.853 × ASM_pred and SMI_recalibrated = 1.371 + 0.738 × SMI_pred. For ASM, the posterior median intercept was 1.537 (95% CrI = 0.793–2.293), and the posterior median slope was 0.853 (95% CrI = 0.815–0.891). For SMI, the posterior median intercept was 1.371 (95% CrI = 0.975–1.769), and the posterior median slope was 0.738 (95% CrI = 0.683–0.795). For both outcomes, the 95% CrIs for the intercept excluded 0 and those for the slope excluded 1, providing evidence of systematic departure from the identity relationship. Together with the positive empirical mean biases of the original predictions, these findings indicated overall overestimation, although the magnitude and direction of the conditional prediction error varied across the prediction range. The ASM_pred calibration slope was closer to 1 than the SMI_pred calibration slope; however, neither original equation demonstrated perfect calibration, and both equations overestimated the corresponding DXA measurements on average (Figure 1).

**FIGURE 1:**
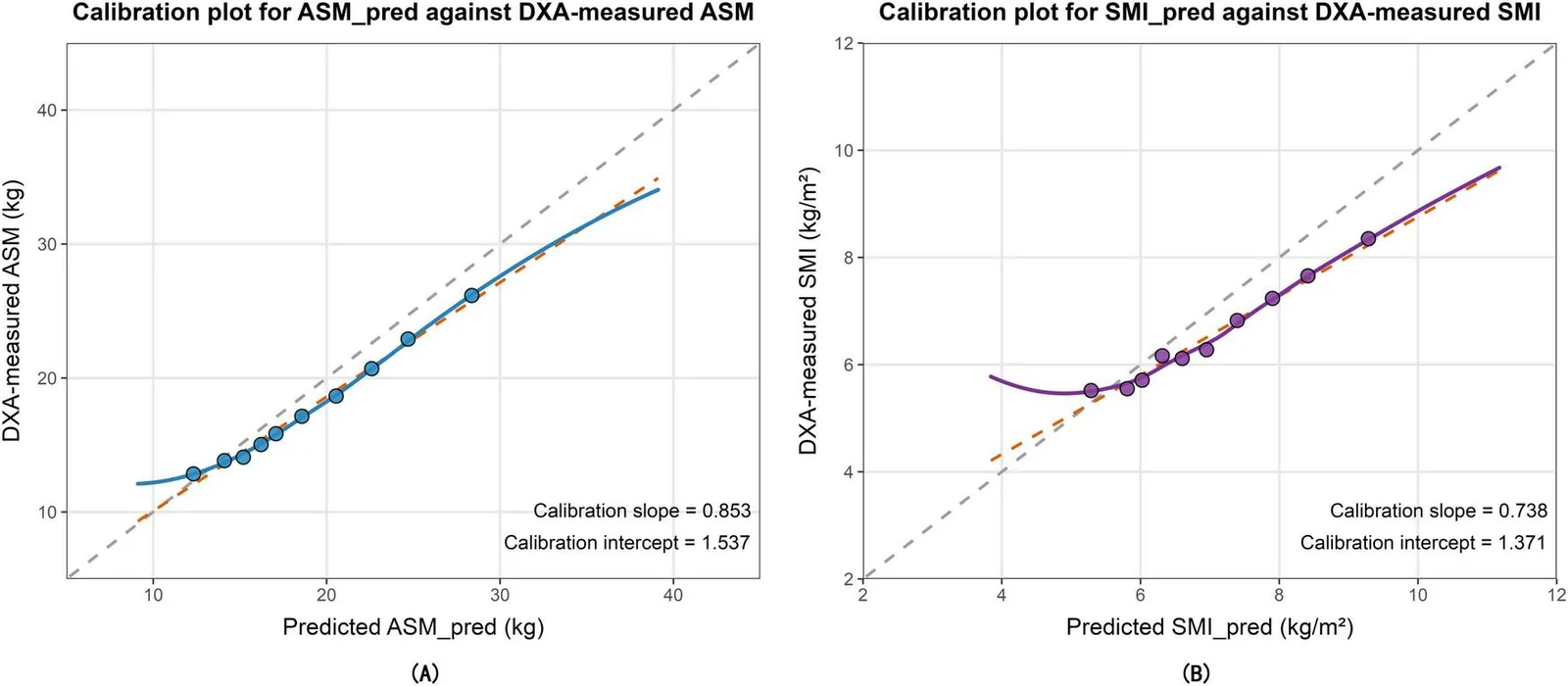
Calibration plots for predicted versus DXA-measured values. Points represent binned observations; solid curves indicate loess-smoothed calibration; dashed lines indicate linear calibration fit; grey diagonal lines indicate the line of identity. **(A)** ASM calibration model. **(B)** SMI calibration model.

Bland–Altman analysis showed that ASM_pred overestimated DXA-measured ASM by a mean of 1.25 kg, with 95% limits of agreement from -3.14 to 5.64 kg. A small but statistically significant positive proportional bias was present, with a regression slope of 0.047 (*P* = 0.028), indicating that the overestimation of ASM tended to increase as the mean ASM level increased. Thus, the equation showed relatively greater overestimation among participants with higher muscle mass (Figure 2A). SMI_pred overestimated DXA-measured SMI by a mean of 0.457 kg/m^2^, with 95% limits of agreement from -1.19 to 2.10 kg/m^2^. There was no evidence of proportional bias, with a regression slope of 0.027 (*P* = 0.419), although substantial individual-level disagreement remained (Figure 2B). Scatterplots and fitted lines supported these findings, showing closer alignment between ASM_pred and ASM than between SMI_pred and SMI (Figure 3). These findings indicate that acceptable performance for continuous ASM estimation did not translate into acceptable transportability of indexed SMI for threshold-based use.

**FIGURE 2:**
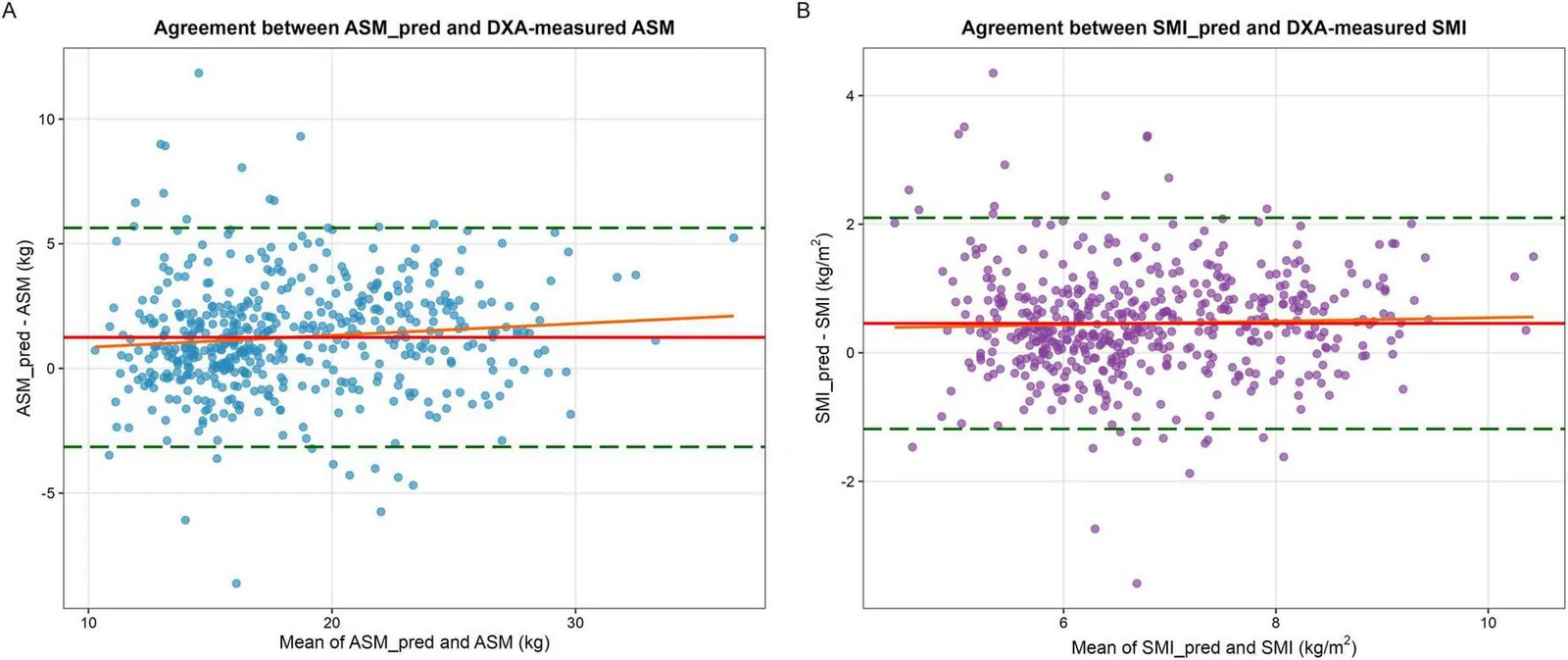
Agreement between predicted and measured values for ASM and SMI (Bland–Altman plots). **(A)** Bland–Altman plot for ASM_pred versus ASM. **(B)** Bland–Altman plot for SMI_pred versus SMI.

**FIGURE 3:**
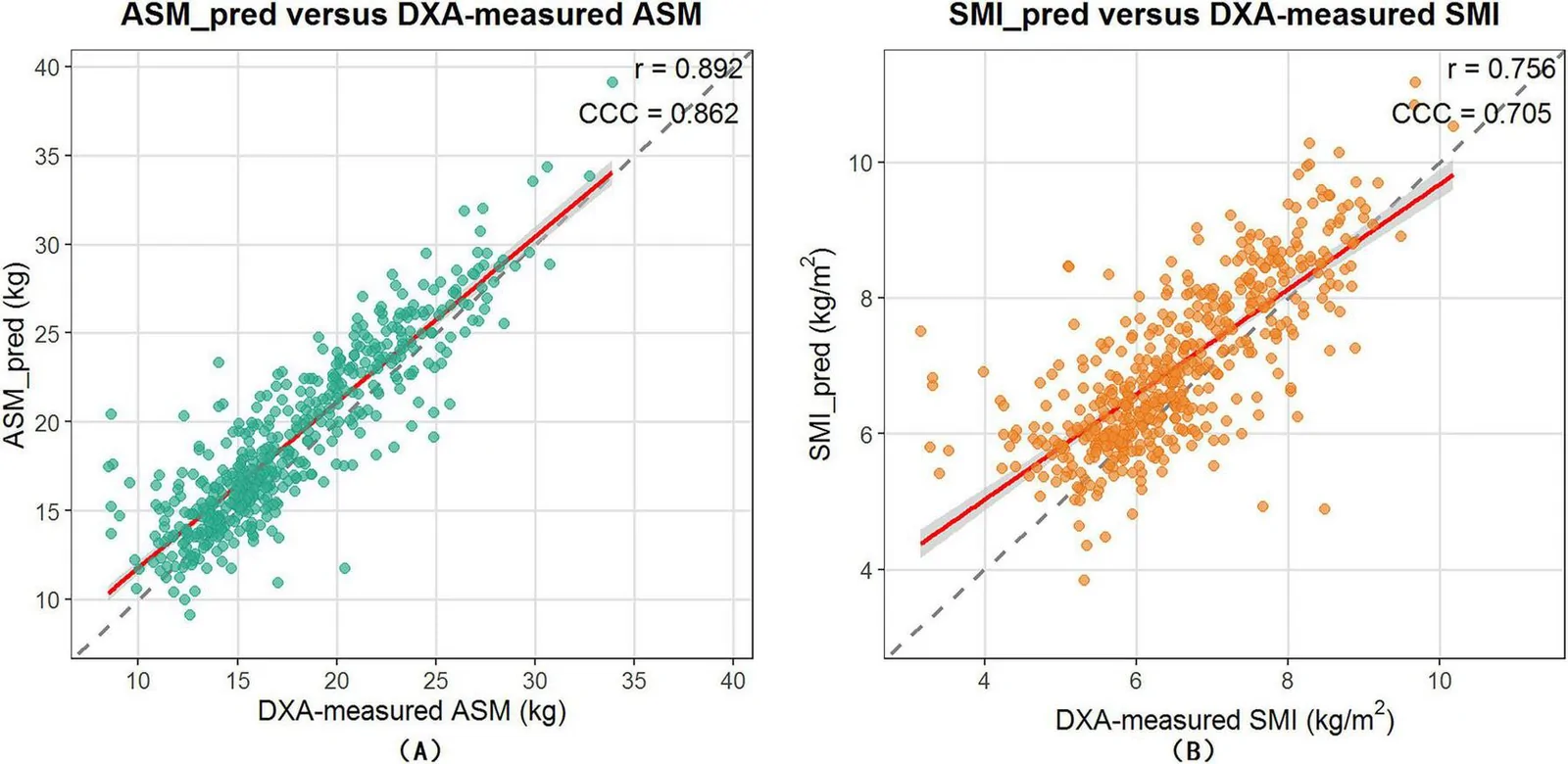
Relationship between predicted and measured values for ASM and SMI (scatter plots with fitted lines). **(A)** Scatter plot and fitted relationship for ASM_pred vs. ASM. **(B)** Scatter plot and fitted relationship for SMI_pred vs. SMI.

In the apparent analysis, Bayesian recalibration reduced ASM MAE from 1.94 to 1.53 kg and RMSE from 2.56 to 2.12 kg, while CCC increased from 0.862 (*95% CI* = 0.843–0.878) to 0.886 (*95% CI* = 0.867–0.903). For SMI, MAE decreased from 0.721 to 0.555 kg/m^2^ and RMSE decreased from 0.954 to 0.775 kg/m^2^, while CCC increased from 0.705 (*95% CI* = 0.667–0.738) to 0.728 (*95% CI* = 0.689–0.761). Mean bias was reduced from 1.25 to 0.0009 kg for ASM and from 0.457 to 0.0003 kg/m^2^ for SMI. The Bayesian 95% posterior predictive intervals covered 474 of 501 DXA-measured ASM values (94.6%) and 472 of 501 DXA-measured SMI values (94.2%), both close to the nominal 95% coverage level. Despite these improvements, the recalibrated estimates retained substantial individual-level uncertainty, as reflected by residual standard deviations of 2.12 kg for ASM (*95% CrI* = 2.00–2.26) and 0.775 kg/m^2^ for SMI (*95% CrI* = 0.731–0.827). The 95% limits of agreement after recalibration were -4.16 to 4.16 kg for ASM and -1.52 to 1.52 kg/m^2^ for SMI. Because the recalibration models were estimated and evaluated in the same sample, these apparent improvements should not be interpreted as independent external validation of the updated equations (Figure 4).

**FIGURE 4:**
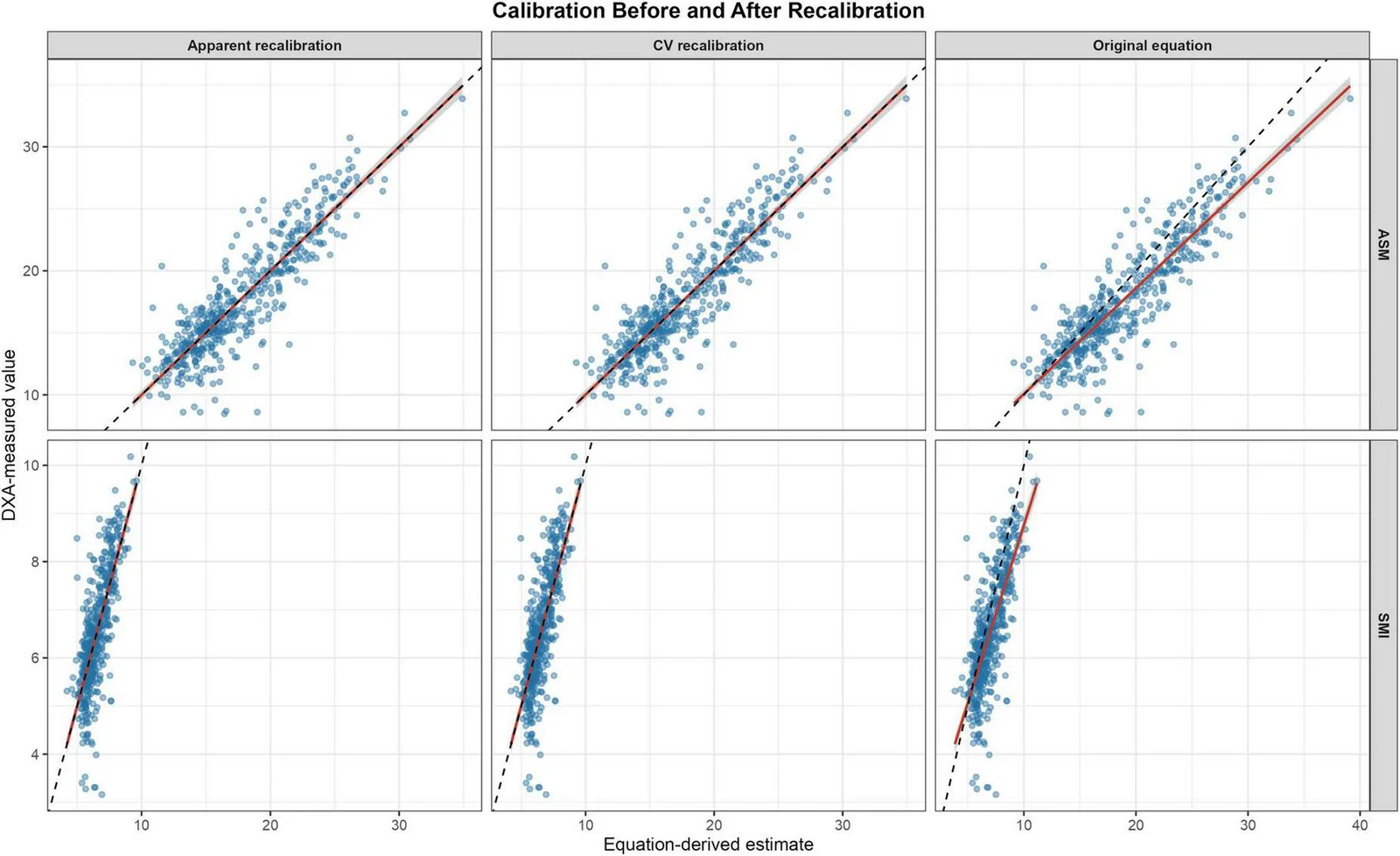
Calibration of the original and recalibrated equations for predicting DXA-measured ASM and SMI.

Exploratory subgroup correlations are presented in the Supplementary material. Several estimates were imprecise, particularly for the sarcopenia subgroup, which included only 31 participants. In this subgroup, the correlation between SMI_pred and SMI was 0.481 (*95% CI* = 0.153–0.714). These estimates should not be interpreted as evidence of subgroup-specific model performance.

Columns show the original equation, apparent recalibration, and repeated 10-fold cross-validated recalibration, respectively; rows show ASM and SMI. The dashed diagonal line represents perfect calibration, and the solid line represents the fitted calibration relationship. Apparent recalibration was estimated and evaluated in the same dataset, whereas cross-validated estimates were obtained from held-out observations.

### 3.3 Classification performance for dXA-defined low muscle mass

When the conventional DXA-based AWGS cut-offs were directly applied to SMI_pred, classification performance for DXA-defined low SMI was poor. The sensitivity was 0.211, whereas specificity was 0.963, indicating marked under-detection. In total, 97 of 123 DXA-defined low-SMI cases were missed, and the estimated prevalence of low SMI was markedly lower using SMI_pred than using DXA (8.0% vs. 24.6%). The PPV was 0.650, the NPV was 0.790, accuracy was 0.778, and Cohen’s kappa was 0.226. The seemingly acceptable overall accuracy mainly reflected high specificity and class imbalance rather than adequate case detection. These findings indicate that direct transfer of DXA-based cut-offs to equation-derived SMI is inappropriate in this cohort.

Bayesian linear recalibration increased the predicted prevalence of low SMI from 8.0% to 16.0% and improved sensitivity from 0.211 to 0.374. However, specificity decreased from 0.963 to 0.910, and 77 of 123 DXA-defined low-SMI cases remained undetected. The corresponding PPV, NPV, accuracy, and Cohen’s kappa were 0.575, 0.817, 0.778, and 0.322, respectively. Thus, recalibration improved case detection and agreement but did not provide adequate classification performance for direct replacement of DXA-based SMI thresholds.

Sex-stratified analyses showed that the improvement in sensitivity after recalibration was more pronounced in males than in females. In males, sensitivity increased from 0.216 to 0.529, while specificity decreased from 0.971 to 0.848. In females, sensitivity increased from 0.208 to 0.264, while specificity decreased from 0.958 to 0.946. These subgroup estimates should be interpreted cautiously because of limited event numbers. Secondary logistic models showed no statistically significant SMI_pred × sex interaction.

### 3.4 Secondary analyses for broader sarcopenia classification

Exploratory models for pre-sarcopenia/sarcopenia versus normal status and sarcopenia versus all other participants are reported in the Supplementary material. BMI was inversely associated with the odds of pre-sarcopenia/sarcopenia in the binary logistic models, whereas age was positively associated with sarcopenia in the sarcopenia-only models. The SMI_pred main effect and the SMI_pred × sex interaction were not statistically significant in these models.

These analyses should be interpreted as exploratory because only 31 sarcopenia events were available. Several subgroup analyses had an events-per-variable ratio below 10, particularly the age-stratified and sex-by-age-stratified sarcopenia models. Therefore, the corresponding estimates may be unstable and should not be used to support definitive subgroup-specific conclusions.

## 4 Discussion

In this independent DXA-referenced external validation study, acceptable performance for absolute ASM estimation did not translate into acceptable transportability of equation-derived SMI for threshold-based classification. Direct application of DXA-based AWGS cut-offs to SMI_pred resulted in substantial under-detection of DXA-defined low muscle mass. From a clinical decision-making perspective, equation-derived SMI and DXA-derived SMI were not interchangeable when classification depended on fixed diagnostic thresholds.

The primary findings can be summarized as follows. First, ASM_pred showed strong correlation and good concordance with DXA-measured ASM, but it retained systematic overestimation, imperfect calibration, and wide individual-level limits of agreement. Second, SMI_pred showed weaker correlation, lower concordance, and clear miscalibration relative to DXA-measured SMI. Third, this translated into clinically important misclassification when fixed DXA-based cut-offs were applied: sensitivity for low muscle mass was only 0.205, and 105 of 132 DXA-defined cases were missed. Fourth, in exploratory secondary sarcopenia-related classification analyses, SMI_pred provided limited incremental information beyond age, sex, and BMI; these findings should be interpreted cautiously because of the small number of sarcopenia events. Taken together, these findings indicate that anthropometric equations may be useful for rough population-level approximation of ASM, but their derived SMI should not be assumed to be interchangeable with DXA for low-muscle-mass classification at fixed diagnostic thresholds. The novelty of the present study lies primarily in testing whether an established DXA-based diagnostic threshold remains valid when transferred to an equation-derived index in an external population. This extends conventional validation based on correlation or average prediction error by directly evaluating calibration near the clinical decision boundary, missed-case rates, and the consequences of measurement error for individual-level classification.

### 4.1 Predicted ASM acceptable; indexing ASM to SMI weakens validity

A key finding was that the same equation performed differently at the ASM and SMI levels. ASM_pred showed a strong correlation with measured ASM, but this strong association coexisted with systematic overestimation, a calibration slope below 1, wide individual-level limits of agreement, and a small positive proportional bias. Thus, the original equation preserved ranking information better for ASM than for SMI, but it was not fully interchangeable with DXA even at the ASM level. Consistent with our findings, one of our previous studies showed that Wen equation-derived ASM was strongly correlated with DXA-measured ASM in both healthy adults and patients with type 2 diabetes mellitus. However, it systematically overestimated ASM and demonstrated only moderate agreement, with wider limits of agreement and poorer classification performance in some subgroups, particularly among women with type 2 diabetes mellitus (11). This suggests that, for absolute appendicular skeletal muscle mass, anthropometric equations can preserve substantial information in relatively healthy adults, consistent with established associations of muscle mass with body weight, age, and sex (12).

However, after ASM_pred was indexed by height^2^ to generate SMI_pred, performance deteriorated: calibration worsened, concordance declined, and discrimination for low muscle mass became clinically inadequate. This pattern is consistent with error amplification at the indexed level. Methodologically, this indicates that acceptable estimation of absolute muscle mass does not guarantee suitability for threshold-based clinical stratification. Because ASM_pred is already constructed from variables including height and weight, further scaling by height^2^ may alter the error structure and amplify systematic error. More broadly, concerns have been raised that height-indexed SMI may not always be the most robust representation of skeletal muscle mass (13). Any such bias may be more pronounced in individuals with atypical stature or body composition. For example, Yee et al. (14), reported that age-related height loss may cause height-based muscle indices to underestimate muscle deficiency in older women. Similarly, Tanaka et al. (15) showed that a modified SMI scaled by maximal lifetime height outperformed conventional SMI in postmenopausal women, particularly among those with height loss. Together, these studies support the possibility that indexation may magnify, rather than attenuate, error in predicted muscle measures and thereby weaken discrimination of true low muscle mass.

Our secondary models should be interpreted as testing whether SMI_pred adds information beyond basic clinical variables, rather than simply whether it is associated with sarcopenia status. In that context, the limited incremental value of SMI_pred suggests that much of its information is already captured by age, sex, and body size. More broadly, these results reinforce an important principle: correlation, even when strong, does not establish interchangeability or threshold transportability (16). For threshold-based use, calibration and classification performance around clinically relevant cut-offs matter more than correlation alone.

### 4.2 Limited discrimination of equation-derived SMI across sarcopenia-related categories

We found that DXA-measured ASM and SMI showed clear differences across the normal, low-muscle-mass-only, and sarcopenia groups, whereas between-group differences for ASM_pred and SMI_pred were markedly attenuated. In particular, between-group separation for ASM_pred and SMI_pred was noticeably weaker and less consistent than for the corresponding DXA-derived indices. This suggests that anthropometric prediction indices may still reflect a general downward trend at the group mean level, but their discriminatory precision becomes insufficient when applied to disease-related stratification. This limitation is especially relevant for identifying individuals with low muscle mass but preserved grip strength, a state that depends heavily on accurate assessment of muscle quantity. Because this state is defined primarily by reduced muscle quantity before overt decline in muscle strength, its identification depends heavily on accurate assessment of muscle mass. If predicted SMI does not adequately approximate true SMI, the earliest populations to be affected are likely to be those in the initial stage of disease evolution, in whom the changes are relatively subtle. For example, Ida et al. (17) reported in a meta-analysis that SARC-F, one of the early screening tools recommended for sarcopenia (6), although highly specific, has poor sensitivity (17).

In exploratory secondary analyses, SMI_pred showed better discrimination for sarcopenia versus all other participants than for pre-sarcopenia or sarcopenia versus normal status. However, these findings should not be interpreted as evidence that SMI_pred can replace DXA for threshold-based identification, particularly given the small number of sarcopenia cases. This further suggests that the prediction framework may be more likely to identify typical sarcopenia cases characterized by older age, lower BMI, and a generally higher overall risk profile, while remaining relatively insensitive to early low muscle mass states. Our classification analysis of DXA-defined low SMI supports this interpretation: the prediction method demonstrated high specificity but very low sensitivity, indicating a tendency to under-identify positive cases and consequently miss a substantial number of true low-muscle-mass cases (18, 19). For a screening tool intended for early detection, this performance pattern is suboptimal because the cost of missing truly low-muscle-mass individuals is usually greater than that of referring some additional individuals for confirmatory evaluation. From a prevention-oriented perspective, this limitation has clear practical implications, as the population most in need of early community-based identification and intervention is often the pre-sarcopenia group rather than those who have already progressed to overt functional impairment (20). Although secondary binary models showed moderate discrimination for broader sarcopenia-related contrasts, this should not be interpreted as evidence that SMI_pred can replace DXA. Risk stratification and direct measurement substitution are different questions, and the primary validation target in this study was transportability relative to DXA.

Furthermore, in both the ordinal Bayesian model and the softmax model, SMI_pred did not show a clear independent association after adjustment for age, BMI, and sex, whereas age and BMI remained consistently informative. This suggests that, in the current sample, the incremental information provided by SMI_pred was limited, and that part of the risk captured by SMI_pred may already have been absorbed by basic variables such as age and BMI, given that the inclusion of covariates can alter coefficient distributions of other variables in the model (21). In other words, because SMI_pred is fundamentally constructed from height, weight, age, and sex, it remains largely driven by those original variables. Therefore, once these variables are already included in the model, SMI_pred may not contribute additional sarcopenia stratification information beyond basic body size characteristics. This issue may be even more relevant in secondary sarcopenia related to chronic diseases, where BMI, anthropometric markers, or biochemical indicators often fail to evaluate sarcopenia accurately (22). These findings also have implications for the development of future screening tools: if the aim is to build a genuinely informative surrogate marker for low muscle mass, reliance on conventional anthropometric parameters alone is unlikely to be sufficient, and variables more directly reflecting muscle structure, function, or body composition distribution may need to be incorporated.

### 4.3 Age and BMI are more stable correlates of sarcopenia stratification

We found that older age was significantly associated with higher sarcopenia grade and risk, whereas higher BMI was associated with lower risk. These findings are consistent with the broader epidemiological literature on sarcopenia and aging-related body composition changes (23, 24). Age is one of the most fundamental determinants of sarcopenia, reflecting multiple biological processes accompanying aging, including declining muscle mass, neuromuscular impairment, reduced protein synthesis, cumulative inflammatory burden, and endocrine alterations (25–27). In our study, the sarcopenia group was substantially older than the other groups, and age remained a stable factor even in multivariable models, indicating that even in a relatively healthy health-examination cohort, aging remains one of the principal background drivers of sarcopenia risk.

The inverse association between BMI and sarcopenia risk should be interpreted cautiously. It more likely reflects the greater susceptibility of individuals with lower body weight or lower nutritional reserve to low muscle mass, rather than implying that higher BMI is intrinsically protective. On the one hand, in generally healthy populations, a lower BMI often indicates reduced overall nutritional reserve and lower lean mass, thereby increasing the likelihood of low muscle mass. On the other hand, BMI cannot distinguish fat mass from lean mass and cannot identify more complex phenotypes such as sarcopenic obesity (28, 29). Accordingly, the observed inverse association should not be interpreted as evidence that higher BMI is biologically protective. Particularly in middle-aged and older adults, higher BMI may coexist with excess adiposity, chronic inflammation, and functional decline (30), and its relationship with sarcopenia may be nonlinear or phenotype-dependent. Future studies should further distinguish among uncomplicated low muscle mass, sarcopenic obesity, and sarcopenia occurring under different fat distribution patterns.

### 4.4 Defining the appropriate role of anthropometric muscle-prediction equations

With the increasing demand for large-scale population studies and community screening, anthropometric equations for predicting muscle mass have been widely used in settings where DXA or BIA is unavailable. Previous studies have frequently highlighted the low cost, easy accessibility, and utility of these equations in epidemiological research, often reporting favorable fit within the original derivation samples (31, 32). However, our findings suggest that being “usable for estimation” does not necessarily mean being “suitable for threshold-based diagnostic stratification” (33). If the purpose is merely to approximate mean ASM at the population level or compare overall between-group differences, ASM_pred may have some practical value. In contrast, if the aim is to identify individuals with low muscle mass according to AWGS criteria, screen for pre-sarcopenia, make individual-level clinical decisions, or use SMI as a key exposure or outcome in analyses that depend on absolute values or fixed thresholds, such equations – particularly the indexed SMI_pred – should not be treated as substitutes for DXA without population-specific validation of calibration and classification performance.

This conclusion has practical implications for both epidemiological research and clinical practice. In the context of large cohort studies, our findings caution against treating anthropometric estimates as interchangeable with DXA-derived indices when the target is threshold-based case identification rather than population-level approximation. In large databases, researchers often use predicted ASM/SMI instead of measured values for feasibility reasons and then estimate sarcopenia prevalence, examine risk factors, or develop prognostic models. Our validation target was deliberately aligned with this real-world practice: we tested direct threshold transfer, not because we endorse it, but because it is common and clinically consequential. However, if the substitute measure is poorly calibrated, this may not only distort prevalence estimates but also attenuate the validity of exposure-outcome associations and, in some circumstances, introduce differential misclassification (34). Clinically, if community screening relies solely on predicted SMI to identify low muscle mass, some truly low-muscle-mass individuals may be missed, while others without low muscle mass may be incorrectly classified, thereby affecting subsequent nutritional, exercise, and follow-up strategies. Our data provide direct external-validation evidence supporting the recent concern that formula-based ASM estimation may be acceptable for rough summarization but can be misleading when used to infer low muscle mass prevalence or sarcopenia status without independent calibration. A more appropriate positioning may therefore be that anthropometric prediction equations can serve as an initial risk-indication tool in resource-limited settings, helping to identify individuals who should undergo further DXA, BIA, muscle strength, or physical performance assessment; however, they should not be regarded as diagnostically equivalent to DXA, particularly not as stand-alone tools for low muscle mass classification or sarcopenia staging.

From the perspective of clinical pathways, the value of a single anthropometric prediction index may lie not in replacing DXA but in being combined with other simple tools. For example, combining SMI_pred with handgrip strength, calf circumference, SARC-F, or simple physical performance tests 6 may be more likely to improve screening sensitivity and reduce missed cases than using any single index alone. Future studies should compare the net benefit of anthropometric prediction alone versus multi-indicator screening strategies across different resource settings.

In the present study, simple intercept-and-slope recalibration reduced MAE and RMSE and removed most of the mean bias in cross-validated analyses. However, residual proportional bias remained, and the recalibrated equations were developed and internally evaluated in the same source cohort. Independent validation in a separate population, together with evaluation of threshold-based sensitivity and specificity, is required before the updated equations can be recommended for clinical classification. In practical terms, equation-derived ASM may remain useful for descriptive population-level analyses, preliminary risk indication, participant ranking, hypothesis generation, and sensitivity analyses in large datasets without direct body-composition measurements, particularly when the equation has been validated or recalibrated in a population similar to the target sample. Equation-derived SMI may also be explored as one component of a multivariable screening strategy, but DXA-based cut-offs should not be transferred to SMI_pred without specific validation of the resulting classification rule. DXA confirmation remains important when an individual diagnosis or sarcopenia classification depends on a fixed muscle-mass threshold, when an estimated value lies near the diagnostic cut-off, when the result will guide treatment, referral, or eligibility decisions, or when small longitudinal changes are interpreted as true changes in muscle mass. Thus, anthropometric prediction equations should be used cautiously as screening or research aids rather than diagnostic substitutes, particularly when the clinical target is threshold-based identification of low muscle mass.

### 4.5 Anthropometric equations based mainly on body size may not capture inter-individual heterogeneity in muscle composition

The bias observed in this study may largely arise from the inherent structural limitations of anthropometric equations. These equations mainly rely on age, height, weight, and sex to estimate muscle mass, and are essentially based on approximating lean tissue quantity through body size characteristics. However, skeletal muscle mass is influenced not only by general body size but also by fat distribution, physical activity, nutritional status, hormonal milieu, inflammatory burden, organ function, and genetic background (35–37). Among individuals with similar body weight, the ratio of fat to muscle, appendicular muscle distribution, and functional reserve may differ substantially (38). Such heterogeneity is difficult to capture accurately using basic anthropometric parameters alone.

In addition, DXA provides relatively direct body composition information, whereas prediction equations generate statistical estimates based on average relationships observed in populations (9). The two approaches are therefore not equivalent in the level or source of information they provide at the individual level. When the target population is similar to the original derivation sample in terms of age structure, body size distribution, adiposity, and health profile, predictive performance may remain acceptable. However, once these characteristics shift in an external sample, both calibration and classification performance may decline substantially, which is a common limitation of anthropometric equations in external applications. In our study, the participants were healthy adults undergoing health examinations, spanning a wide age range from younger adulthood to old age. Because trajectories of muscle loss, fat infiltration, and body composition relations differ across age stages (39), the stability of a single equation across the entire adult life course may be inherently limited.

### 4.6 Strengths and limitations

This study has several notable strengths. First, we used DXA as the reference method to externally validate the anthropometric prediction indices, thereby enhancing the clinical interpretability of the findings. Because DXA-based body-composition measurements were available for all included participants, the study enabled direct evaluation of agreement, calibration, and threshold-based classification performance. The small number of sarcopenia events also resulted in an EPV below 10 for the overall sarcopenia-versus-others model and substantially lower EPVs after stratification. Accordingly, subgroup and secondary regression estimates were exploratory, potentially unstable, and were not used as primary evidence. Second, rather than limiting validation to conventional prediction accuracy or correlation, we specifically evaluated agreement, calibration, threshold transportability, and individual-level classification performance. This design allowed us to assess whether equation-derived SMI could preserve clinically meaningful DXA-based decisions, which represents the principal methodological contribution of the study. Third, we used AWGS 2019 criteria, enhancing the clinical relevance of the findings for East Asian practice. Fourth, we used a Bayesian framework for the continuous calibration models to obtain direct posterior probability distributions for the calibration intercept and slope, allowing uncertainty in departures from the identity values of 0 and 1 to be quantified jointly. Weakly informative priors were selected to regularize estimation without materially dominating the likelihood, and convergence and posterior predictive diagnostics were examined. This choice was made for uncertainty quantification rather than to alter the estimand or compensate for limited model fit.

Several limitations should also be acknowledged. First, this was a single-center retrospective study based on individuals undergoing hospital-based health examinations and DXA testing, and selection bias may therefore exist. In addition, the predefined exclusion of participants with diabetes, malignancy, cirrhosis, severe cardiovascular or cerebrovascular disease, and other conditions affecting muscle mass restricted the cohort to a relatively healthy subset. This restriction may have reduced clinical heterogeneity and yielded more favorable performance estimates than would be expected in an unselected health-examination or clinical population. The representativeness of the sample is therefore limited, and the findings should not be extrapolated directly to the general community, rural populations, specific disease groups, or patients with chronic or muscle-wasting conditions. External validation is needed across different geographic regions, ethnic populations, and clinical settings, including community and primary-care populations, hospitalized patients, and individuals with chronic or muscle-wasting conditions. Such studies should also examine whether differences in body composition, age distribution, disease burden, and DXA manufacturer or analysis software affect calibration and threshold-based classification performance. Second, although the cohort covered a broad adult age range, the number of participants with sarcopenia was relatively small, which limits precision for secondary regression analyses. Third, the equation evaluated here was a specific anthropometric prediction formula developed for a Chinese population, and our results mainly reflect the external applicability of this formula in the current sample; they should not be generalized directly to other ASM prediction models. In addition, validation was performed using a single DXA platform (GE Lunar Prodigy), and the findings may not fully generalize across DXA manufacturers, software versions, or processing pipelines. Accordingly, our findings should be interpreted as evidence regarding one widely used anthropometric equation rather than a rejection of all non-DXA muscle-mass surrogates. Fourth, low muscle mass was defined on the basis of DXA and low muscle strength was assessed using handgrip strength, but physical performance indicators such as gait speed or chair stand time were not included. Therefore, the study could not identify severe sarcopenia or evaluate the complete AWGS 2019 sarcopenia spectrum. Fifth, although several diseases known to clearly affect muscle mass were excluded, residual confounding cannot be ruled out in this retrospective design. Potentially relevant factors such as physical activity, diet and nutrition, menopausal status, inflammatory markers, and social or behavioral factors were not measured (40, 41), and all of these may influence both muscle mass and prediction bias. Additionally, hydration status, which can subtly alter body weight and thus the equation’s output without reflecting true muscle mass changes, was not systematically controlled. Finally, this study primarily assessed cross-sectional measurement validity and cannot determine the longitudinal value of predicted indices for tracking changes in muscle mass, predicting adverse outcomes, or monitoring intervention responses.

### 4.7 Future directions

Future studies should evaluate simple recalibration or threshold remapping of equation-derived SMI, compare multiple anthropometric equations across geographically and clinically diverse Chinese populations, and extend external validation to other ethnic populations and healthcare settings. These studies should determine whether population-specific differences in body composition and disease burden require new coefficients or locally derived classification thresholds and should test whether multi-indicator screening strategies can improve sensitivity in resource-limited settings. Second, incorporating additional variables such as body circumferences, waist-to-hip ratio, calf circumference, physical activity, nutritional markers, biochemical indicators, or ultrasound-based muscle thickness may help develop new surrogate tools with both accessibility and improved accuracy. Third, prospective cohort studies are needed to evaluate whether predicted ASM/SMI can predict clinically relevant outcomes such as falls, hospitalization, disability, and mortality, to determine whether these indices may still have value as risk stratification tools even if they cannot precisely replace DXA. Fourth, for the key window of pre-sarcopenia, more sensitive early screening algorithms should be developed to avoid missing opportunities for timely intervention because of measurement error. Finally, because the present study focused mainly on cross-sectional validity, future work should also examine the long-term value of these predicted indices in monitoring muscle mass trajectories, forecasting adverse outcomes, and assessing responses to interventions.

The present study should not be interpreted as challenging the utility of anthropometric equations for population-level approximation, but rather as clarifying that their use for individual-level case identification requires independent validation and, where possible, population-specific recalibration or threshold remapping performed before clinical implementation.

## 5 Conclusion

In this independent DXA-referenced external validation study of relatively healthy Chinese adults undergoing health examinations, the Wen equation preserved strong correlation for absolute ASM but showed systematic bias and imperfect agreement. Equation-derived SMI demonstrated poorer calibration and very low sensitivity when DXA-based AWGS cut-offs were transferred directly. Simple recalibration improved continuous prediction within this cohort but did not eliminate residual proportional bias and requires independent validation, including evaluation of low-muscle-mass classification. The original or recalibrated equations should therefore not be regarded as stand-alone substitutes for DXA when clinical decisions depend on fixed diagnostic thresholds.

## Data Availability

The original contributions presented in the study are included in the article/Supplementary material, further inquiries can be directed to the corresponding author.

## References

[1] Guridi M Kupr B Romanino K Lin S Falcetta D Tintignac L et al. Alterations to mTORC1 signaling in the skeletal muscle differentially affect whole-body metabolism. *Skelet Muscle*. (2016) 6:13. 10.1186/s13395-016-0084-8 27004103 PMC4800774

[2] Afzali AM Müntefering T Wiendl H Meuth SG Ruck T. Skeletal muscle cells actively shape (auto)immune responses. *Autoimmun Rev*. (2018) 17:518–29. 10.1016/j.autrev.2017.12.005 29526638

[3] Narici M McPhee J Conte M Franchi MV Mitchell K Tagliaferri S et al. Age-related alterations in muscle architecture are a signature of sarcopenia: the ultrasound sarcopenia index. *J Cachexia Sarcopenia Muscle*. (2021) 12:973–82. 10.1002/jcsm.12720 34060717 PMC8350200

[4] Beaudart C Rizzoli R Bruyère O Reginster JY Biver E. Sarcopenia: burden and challenges for public health. *Arch Public Health*. (2014) 72:45. 10.1186/2049-3258-72-45 25810912 PMC4373245

[5] Lee JH Kim HJ Han S Park SJ Sim M Lee KH. Reliability and agreement assessment of sarcopenia diagnosis through comparison of bioelectrical impedance analysis and dual-energy x-ray absorptiometry. *Diagnostics* . (2024) 14:899. 10.3390/diagnostics14090899 38732314 PMC11083379

[6] Chen LK Woo J Assantachai P Auyeung TW Chou MY Iijima K et al. Asian working group for sarcopenia: 2019 consensus update on sarcopenia diagnosis and treatment. *J Am Med Dir Assoc.* (2020) 21:300e–7e. 10.1016/j.jamda.2019.12.012 32033882

[7] Zanker J Sim M Anderson K Balogun S Brennan-Olsen SL Dent E et al. Consensus guidelines for sarcopenia prevention, diagnosis and management in Australia and New Zealand. *J Cachexia Sarcopenia Muscle*. (2023) 14:142–56. 10.1002/jcsm.13115 36349684 PMC9891980

[8] Abdalla PP da Silva LSL Venturini ACR Júnior MFT Schneider G Dos Santos AP et al. Anthropometric equations to estimate appendicular muscle mass from dual-energy x-ray absorptiometry (DXA): a scoping review. *Arch Gerontol Geriatr*. (2023) 110:104972. 10.1016/j.archger.2023.104972 36893510

[9] Wen X Wang M Jiang CM Zhang YM. Anthropometric equation for estimation of appendicular skeletal muscle mass in Chinese adults. *Asia Pac J Clin Nutr.* (2011) 20:551–6. 22094840

[10] Liu M Wang J Yang C Sun G. Estimation of appendicular skeletal muscle mass in studies based on CHARLS may cause unreliable conclusion. *J Cachexia Sarcopenia Muscle*. (2025) 16:e13800. 10.1002/jcsm.13800 40241386 PMC12003915

[11] Liu Z Ren Y Wang Y Zhao X Cao Y. Evaluation of the agreement and clinical utility of anthropometric equation-estimated appendicular skeletal muscle mass compared to dual-energy x-ray absorptiometry in patients with type 2 diabetes mellitus. *Clin Nutr ESPEN*. (2026) 73:103315. 10.1016/j.clnesp.2026.103315 42097506

[12] Gallagher D Heymsfield SB. Muscle distribution: variations with body weight, gender, and age. *Appl Radiat Isot*. (1998) 49:733–4. 10.1016/s0969-8043(97)00096-1 9569594

[13] Giai J Veloso M Vilotitch A Charrière K Bétry C. Questioning skeletal muscle index for muscle mass reduction assessment in computed tomography: why square the height? *Clin Nutr*. (2025) 52:1–7. 10.1016/j.clnu.2025.07.010 40683130

[14] Yee ML Einoder S Strauss BJG Gilfillan C. Evaluation of an alternative skeletal muscle index for skeletal muscle mass assessment in a group of Australian women. *Age Ageing*. (2022) 51:afac002. 10.1093/ageing/afac002 35150589 PMC9160878

[15] Tanaka KI Morisato Y Nakajima H Kanasaki K Sugimoto T Kanazawa I. Which is a better skeletal muscle mass index for the evaluation of physical abilities: the present height or maximum height? *Intern Med*. (2021) 60:1191–6. 10.2169/internalmedicine.5792-20 33229805 PMC8112974

[16] Engelmann MD. Sammenhaeng eller overensstemmelse mellem kliniske målemetoder? [association or agreement between clinical measurement methods?]. *Ugeskr Laeger.* (2000) 162:4400–1. 10962965

[17] Ida S Kaneko R Murata K. SARC-F for screening of sarcopenia among older adults: a meta-analysis of screening test accuracy. *J Am Med Dir Assoc*. (2018) 19:685–9. 10.1016/j.jamda.2018.04.001 29778639

[18] Obuchowski NA. Receiver operating characteristic curves and their use in radiology. *Radiology*. (2003) 229:3–8. 10.1148/radiol.2291010898 14519861

[19] Smithson M. The receiver operating characteristic area under the curve (or mean ridit) as an effect size. *Psychol Methods*. (2025) 30:678–86. 10.1037/met0000601 37439716

[20] Li YH Wang XH Ya S Jiaoling H Hua N. The optimal cut-off value of five-time chair stand test for assessing sarcopenia among Chinese community-dwelling older adults. *J Cachexia Sarcopenia Muscle*. (2024) 15:756–64. 10.1002/jcsm.13441 38342700 PMC10995269

[21] Chang CR Song Y Li F Wang R. Covariate adjustment in randomized clinical trials with missing covariate and outcome data. *Stat Med*. (2023) 42:3919–35. 10.1002/sim.9840 37394874 PMC12094063

[22] Kim HY Jang JW. Sarcopenia in the prognosis of cirrhosis: going beyond the MELD score. *World J Gastroenterol*. (2015) 21:7637–47. 10.3748/wjg.v21.i25.7637 26167066 PMC4491953

[23] Priyadarsini N Nanda P Devi S Mohapatra S. Sarcopenia: an age-related multifactorial disorder. *Curr Aging Sci*. (2022) 15:209–17. 10.2174/1874609815666220304194539 35249518

[24] Dodds RM Granic A Davies K Kirkwood TB Jagger C Sayer AA. Prevalence and incidence of sarcopenia in the very old: findings from the Newcastle 85+ Study. *J Cachexia Sarcopenia Muscle*. (2017) 8:229–37. 10.1002/jcsm.12157 27897431 PMC5377385

[25] Delbono O Rodrigues ACZ Bonilla HJ Messi ML. The emerging role of the sympathetic nervous system in skeletal muscle motor innervation and sarcopenia. *Ageing Res Rev*. (2021) 67:101305. 10.1016/j.arr.2021.101305 33610815 PMC8049122

[26] Dalle S Rossmeislova L Koppo K. The role of inflammation in age-related sarcopenia. *Front Physiol*. (2017) 8:1045. 10.3389/fphys.2017.01045 29311975 PMC5733049

[27] Miller BF Baehr LM Musci RV Reid JJ Peelor FF Hamilton KL et al. Muscle-specific changes in protein synthesis with aging and reloading after disuse atrophy. *J Cachexia Sarcopenia Muscle*. (2019) 10:1195–209. 10.1002/jcsm.12470 31313502 PMC6903438

[28] Benton MJ Silva-Smith AL. Accuracy of body mass index versus lean mass index for prediction of sarcopenia in older women. *J Frailty Aging*. (2018) 7:104–7. 10.14283/jfa.2018.1 29741194

[29] Müller MJ Braun W Enderle J Bosy-Westphal A. Beyond BMI: conceptual issues related to overweight and obese patients. *Obes Facts*. (2016) 9:193–205. 10.1159/000445380 27286962 PMC5644873

[30] Tay J Goss AM Locher JL Ard JD Gower BA. Physical function and strength in relation to inflammation in older adults with obesity and increased cardiometabolic risk. *J Nutr Health Aging*. (2019) 23:949–57. 10.1007/s12603-019-1260-4 31781724 PMC6996491

[31] Luo YX Zhou XH Heng T Yang LL Zhu YH Hu P et al. Bidirectional transitions of sarcopenia states in older adults: The longitudinal evidence from CHARLS. *J Cachexia Sarcopenia Muscle*. (2024) 15:1915–29. 10.1002/jcsm.13541 39001569 PMC11446714

[32] Huang Y Wang Q Wang G Ding M You Y Li F et al. Cross-sectional associations between physical activity and sarcopenia at different life stages: evidence from CHARLS. *BMC Geriatr*. (2026) 26:153. 10.1186/s12877-025-06938-x 41485020 PMC12866481

[33] Foreman MD. Screening is not the same as diagnosis. *J Gerontol Nurs.* (2006) 32:4–5. 10.3928/00989134-20060601-01 16773855

[34] Rogan WJ Gladen B. Estimating prevalence from the results of a screening test. *Am J Epidemiol*. (1978) 107:71–6. 10.1093/oxfordjournals.aje.a112510 623091

[35] Lipina C Hundal HS. Lipid modulation of skeletal muscle mass and function. *J Cachexia Sarcopenia Muscle*. (2017) 8:190–201. 10.1002/jcsm.12144 27897400 PMC5377414

[36] Rezuş E Burlui A Cardoneanu A Rezuş C Codreanu C Pârvu M et al. Inactivity and skeletal muscle metabolism: a vicious cycle in old age. *Int J Mol Sci*. (2020) 21:592. 10.3390/ijms21020592 31963330 PMC7014434

[37] Prior SJ Roth SM Wang X Kammerer C Miljkovic-Gacic I Bunker CH et al. Genetic and environmental influences on skeletal muscle phenotypes as a function of age and sex in large, multigenerational families of African heritage. *J Appl Physiol.* . (2007) 103:1121–7. 10.1152/japplphysiol.00120.2007 17656630 PMC2811418

[38] Lee JJ Woodard GA Gianaros PJ Barinas-Mitchell E Tepper PG Conroy MB. Ectopic adiposity is associated with autonomic risk factors and subclinical cardiovascular disease in young adults. *Obesity.* (2015) 23:2030–6. 10.1002/oby.21138 26333626 PMC4587338

[39] Holsgrove-West RK Słowiński PM Svensen E Koscien C Fulford J Revuelta Iniesta R et al. Ultrasound predicts skeletal muscle fat infiltration in healthy middle-aged and young adults: validation against MRI. *Exp Physiol*. (2026) 111:2135–47. 10.1113/EP092865 41778905 PMC13140627

[40] Naworska B Brzêk A Ba̧k-Sosnowska M. The relationship between health status and social activity of perimenopausal and postmenopausal women (health status and social relationships in menopause). *Int J Environ Res Public Health*. (2020) 17:8388. 10.3390/ijerph17228388 33198407 PMC7696753

[41] Roubenoff R. Physical activity, inflammation, and muscle loss. *Nutr Rev*. (2007) 65:S208–12. 10.1111/j.1753-4887.2007.tb00364.x 18240550

