# Supplementary material for: Equation-derived SMI not generalizable for DXA-defined low muscle mass in Chinese adults: external validation study
Source: Front Nutr. 2026 Sep 22;13:1887277. doi: 10.3389/fnut.2026.1887277 (PMC13638400; doi:10.3389/fnut.2026.1887277)
Supplement: Supplementary file 1 [file Supplementary_file_1.doc]

**Supplementary Material: Additional Results**

**S1. Cross-validated performance of recalibrated ASM and SMI**

Bayesian linear recalibration models were fitted separately for ASM and SMI. Visual inspection of the posterior draw sequences showed stable mixing without evident temporal trends, and the corresponding posterior distributions were unimodal and well defined (Supplementary Figures S1 and S2). These findings indicated satisfactory computational convergence of the recalibration models. Repeated 10-fold cross-validation was used to assess the performance of recalibration in held-out observations. These cross-validated results were consistent with the apparent recalibration analysis reported in the main text. Recalibration improved average agreement, but the remaining prediction errors were substantial relative to the clinical differences relevant to individual-level classification. Thus, the improvements in aggregate performance did not establish interchangeability between equation-derived and DXA-measured values.

**Supplementary Figure S1. Posterior sampling diagnostics and parameter distributions for the ASM recalibration model**

**Supplementary Figure S2. Posterior sampling diagnostics and parameter distributions for the SMI recalibration model**

**S2. ROC analysis of predicted SMI**

For discrimination between participants with pre-sarcopenia or sarcopenia and those with normal status, SMI_pred had an area under the receiver operating characteristic curve (AUC) of 0.622 (*95% CI*=0.565–0.679). The data-derived cutoff was 6.124 kg/m², corresponding to a sensitivity of 45.5% and a specificity of 75.4%.

For discrimination between participants with sarcopenia and all other participants, the AUC was 0.592 (*95% CI*=0.483–0.700). The data-derived cutoff was 5.453 kg/m², with a sensitivity of 25.8% and a specificity of 94.9%. The confidence interval for this AUC included 0.50, indicating no clear evidence of discrimination beyond chance.

Linear recalibration did not alter the AUC or the sensitivity and specificity achieved at the corresponding ROC-optimal operating points because a positive linear transformation preserves the ranking of individual predictions. Recalibration changed only the numerical values of the cutoffs: from 6.124 to 5.895 kg/m² for pre-sarcopenia or sarcopenia and from 5.453 to 5.400 kg/m² for sarcopenia alone. Overall, SMI_pred showed limited discrimination for the broader outcome and poor discrimination for sarcopenia alone (Recalibrated cutoffs were calculated using the full-precision posterior median coefficients; therefore, minor differences may arise when calculations are reproduced using the rounded coefficients reported in the main text.).(See Supplementary Figure S3)

**Supplementary Figure S3. Receiver operating characteristic curves of predicted SMI for sarcopenia-related outcomes**

**S3. Binary logistic regression analyses**

For pre-sarcopenia or sarcopenia versus normal status, BMI was inversely associated with the outcome in Model 1 (*OR*=0.659; *95% CI*=0.483–0.857; *P*=0.004). SMI_pred was not independently associated with the outcome (*OR*=1.736; *95% CI*=0.466–8.252; *P*=0.444), and neither age nor sex was statistically significant.

After inclusion of the SMI_pred-by-sex interaction in Model 2, BMI remained inversely associated with the outcome (*OR*=0.663; *95% CI*=0.494–0.858; *P*=0.003), whereas the interaction was not statistically significant (*OR*=1.637; *95% CI*=0.760–3.616; *P*=0.213). Model fit did not improve after inclusion of the interaction (AIC, 482.29 for Model 2 versus 481.87 for Model 1).

For sarcopenia versus all other participants, older age was associated with higher odds of sarcopenia in Model 1 (*OR per year*=1.172; *95% CI*=1.095–1.268; *P*<0.001), whereas BMI was inversely associated with sarcopenia (*OR*=0.474; *95% CI*=0.221–0.906; *P*=0.044). SMI_pred was not independently associated with sarcopenia (*OR*=14.849; *95% CI*=0.562–689.740; *P*=0.150). The wide confidence interval indicated substantial statistical uncertainty.

In Model 2, age (*OR per year*=1.177; *95% CI*=1.097–1.277; *P*<0.001) and BMI (*OR*=0.450; *95% CI*=0.200–0.892; *P*=0.043) remained statistically significant. The SMI_pred-by-sex interaction was not significant (*OR*=0.738; *95% CI*=0.198–2.674; *P*=0.643), and the interaction model had a higher AIC than Model 1 (178.08 versus 176.29).

**S4. Multinomial logistic regression analyses**

Using the normal group as the reference category, multinomial logistic regression was used to model pre-sarcopenia and sarcopenia simultaneously. In Model 1, BMI was inversely associated with pre-sarcopenia versus normal status (RRR=0.716; *95% CI*=0.536–0.957; *P*=0.024). SMI_pred, age, and sex were not statistically significant for this comparison.

For sarcopenia versus normal status, older age was associated with sarcopenia (RRR per year=1.173; *95% CI*=1.088–1.264; *P*<0.001), whereas BMI was inversely associated with sarcopenia (RRR=0.430; *95% CI*=0.206–0.895; *P*=0.024). SMI_pred was not independently associated with sarcopenia (RRR=16.856; *95% CI*=0.422–673.137; *P*=0.133).

In Model 2, BMI remained associated with both pre-sarcopenia (RRR=0.716; *95% CI*=0.540–0.949; *P*=0.020) and sarcopenia (RRR=0.419; *95% CI*=0.194–0.906; *P*=0.027). Age also remained associated with sarcopenia (RRR per year=1.175; *95% CI*=1.088–1.269; *P*<0.001). The SMI_pred-by-sex interaction was not statistically significant for either pre-sarcopenia (*P*=0.190) or sarcopenia (*P*=0.963). Model 2 did not improve overall fit (AIC, 583.29 versus 581.15 for Model 1).

**S5. Sex- and age-stratified agreement analyses**

Agreement analyses stratified by sex and age consistently showed stronger performance for ASM than for SMI. Among men, the correlation between ASM_pred and DXA-measured ASM was 0.811 (*95% CI*=0.756–0.855), compared with 0.633 (*95% CI*=0.539–0.712) for SMI_pred and DXA-measured SMI. Among women, the corresponding correlations were 0.617 (*95% CI*=0.543–0.682) for ASM and 0.367 (*95% CI*=0.267–0.460) for SMI. In the overall sample, the correlation was significantly stronger for ASM than for SMI, with a paired bootstrap correlation difference of 0.136 (*95% CI*=0.111–0.164). (See Supplementary Figure S4)

Among participants aged 18–39, 40–59, and ≥60 years, the correlations for ASM were 0.899 (*95% CI*=0.856–0.929), 0.920 (*95% CI*=0.897–0.938), and 0.834 (*95% CI*=0.782–0.875), respectively. The corresponding correlations for SMI were 0.803 (*95% CI*=0.726–0.860), 0.820 (*95% CI*=0.770–0.859), and 0.629 (*95% CI*=0.528–0.712), respectively.(See Supplementary Figure S5)

Agreement for SMI was weakest among women (CCC, 0.327) and among participants aged ≥60 years (CCC, 0.600). A statistically significant proportional bias was also observed for SMI among women (*P*=0.001). These subgroup findings should be interpreted as exploratory because no formal adjustment was made for the multiple subgroup comparisons. Within the sarcopenia subgroup, the correlation between SMI_pred and DXA-measured SMI was 0.481 (*95% CI*=0.153–0.714). This subgroup included only 31 participants, resulting in a wide confidence interval and limited precision. Therefore, this estimate should not be interpreted as reliable evidence of model performance specifically among participants with sarcopenia.

Grip strength distributions differed across the normal, pre-sarcopenia, and sarcopenia groups, with the lowest values observed among participants with sarcopenia (Supplementary Figure S6).

**Supplementary Figure S4. Paired bootstrap distribution of the difference between ASM and SMI prediction correlations**

**Supplementary Figure S5. Subgroup correlations between equation-derived and DXA-measured ASM and SMI**

**Supplementary Figure S6. Distribution of grip strength according to sarcopenia status**

**S6. Sample-size and model-stability considerations**

Only 31 participants (6.2% of the study population) were classified as having sarcopenia. Consequently, regression analyses using sarcopenia alone as the outcome had limited effective sample sizes. The overall sarcopenia-versus-others Model 1 included four predictor variables and had 7.75 events per predictor, excluding the intercept.

The sex- and age-stratified analyses were even more sparse. For sex-stratified models, the number of predictors and the corresponding events-per-predictor ratios were calculated separately; these analyses remained sparse and should be interpreted cautiously. Only two participants aged 18–39 years and four participants aged 40–59 years had sarcopenia, and most sex-by-age strata contained between one and 13 sarcopenia events.

Accordingly, the large ORs and RRRs and their wide confidence intervals, particularly those for SMI_pred and sex, are most plausibly explained by sparse-data instability and should not be interpreted as evidence of strong associations. Similarly, the absence of a statistically significant SMI_pred-by-sex interaction indicates that an interaction was not detected in this sample; it does not establish that no interaction exists.

**S7. Summary of additional analyses**

The additional analyses supported the main findings. Cross-validated recalibration improved average prediction error and concordance but did not eliminate clinically important individual-level disagreement. SMI_pred showed limited rank-based discrimination for pre-sarcopenia or sarcopenia and poor discrimination for sarcopenia alone. In the binary and multinomial models, age and BMI were more consistently associated with sarcopenia-related outcomes than SMI_pred, and inclusion of an SMI_pred-by-sex interaction did not improve model fit. Because the number of sarcopenia events was small, the regression and subgroup findings should be regarded as exploratory.
